# MicroRNA-181a-5p Regulates Inflammatory Response of Macrophages in Sepsis

**DOI:** 10.1515/med-2019-0106

**Published:** 2019-11-20

**Authors:** Zheng Huang, Hang Xu

**Affiliations:** 1 Department of Critical Care Medicine, The First Affiliated Hospital of Shihezi University, No. 107 North 2nd Road, Shihezi 832000, China

**Keywords:** MicroRNA-181a-5p, SIRT, macrophages, sepsis

## Abstract

The aim of this study was to evaluate the role of miR-181a-5p in sepsis, and to further explore the molecular mechanism. RAW 264.7 cells were stimulated with 1 μg/ml LPS for 4 hours. Firstly, qRT-PCR and ELISA was adopted to evaluate the expression of miR-181a-5p and p ro-inflammatory cytokines in RAW 264.7 macrophages a fter LPS stimulation. Results showed that pro-inflammatory cytokines and miR-181a-5p were significantly increased after LPS treatment. Then, we identified that sirtuin-1 (SIRT1) was a direct target of miR-181a-5p and it was down-regulated in LPS treated RAW264.7 macrophages. Furthermore, the data suggested that the miR-181a-5p inhibitor significantly inhibited LPS enhanced inflammatory cytokines expression and NF-κB pathway activation, and these changes were eliminated by SIRT1 silencing. Moreover, the role of the miR-181a-5p inhibitor on sepsis was studied in vivo. We found that the miR-181a-5p inhibitor significantly decreased the secretion of inflammatory factors, and the levels of creatine (Cr), blood urea nitrogen (BUN), aspartate aminotransferase (AST) and alanine aminotransferase (ALT) in a serum for mice with sepsis. However, all the effects were reversed by SIRT1-siRNA. In summary, these results indicated that miR-181a-5p was involved in sepsis through regulating the inflammatory response by targeting SIRT1, suggesting that miR-181a-5p may be a potential target for the treatment of sepsis.

## 1 Introduction

Sepsis is a serious systemic inflammatory response syndrome caused by a dy sregulated response to infection and the process is complex [1, 2]. Sepsis can result in multiple or gan failure and shock, including ca rdiac dysfunction, brain injury, lung damage and liver injury [3, 4, 5]. The high mortality and morbidity rates of sepsis has continued to rise [6]. Despite marked efforts that have been made on systemic inflammation to improve the diagnosis and therapy of sepsis, the molecular mechanisms that contribute to inflammation and subsequent organ injury are not fully understood.

Mi croRNAs (miRNAs) are a group of endogenous non-encoding small molecules with 20-22 nucleotide, that can change the expression and translation of their target genes by bi nding to the 3’untranslated region of target mRNAs [7, 8]. Moreover, several studies have indicated that re strained translational and silenced target genes could treat complex human disease [9, 10]. Importantly, many reports have revealed that mi RNAs also widely distribute in the blood circulation and body fluids [11, 12]. Moreover, miRNAs have been reported as important regulators for inflammation by the regulation of NF-κB signal pathways and may be a potential sepsis bio-marker [13]. In addition, multiple miRNAs including miRNA-143, miR-NA-23a and miR NA-214 have been found to be abnormal in sepsis, which provides central guidance to the diagnosis and treatment of sepsis [14, 15, 16]. MiR -181a-5p, which belongs to miR-181s family, plays an important role in malignant dis eases [17, 18]. MiR-181a-5p has been suggested as a tumor suppressor gene in many cancers [19]. However, whether mi R-181a-5p is involved in the development of sepsis remains unclear.

Previous reports have shown that mac rophages were used as immune cells involved in inflammatory response and played the role of immune suppression in sep sis [20]. Lipopolysaccharide (LPS) is widely used to stimulate inflammation in *in vitro* or *in vivo* models [21]. The stimulation of LPS could result in many intracellular activities in macrophages, including the activation of p65. The activated p65 (p-p65) could transfer into the nucleus and exert the function of inflammation [22].

Sirtuin-1 (SIRT1), a protein deacetylase, is directly implicated in the modulation of inflammatory response and plays an important role in various metabolic and pathophysiological processes, such as inflammation, endothelial function and metabolic disorders [23]. However, little information is available on the relationship between miR-181a-5p and SIRT1 in sepsis *in vivo* or *in vitro*.

In this study, we aimed to investigate the role and underlying mechanisms of miR-181a-5p in sepsis. Our results may provide a potential novel therapeutic target for treating sepsis.

## 2 Materials and methods

### 2.1 Animals

60 male C57BL/6 mice aged 8–10 weeks (20–22g) were obtained from the Experimental Animal Center of FMMU. All animals were housed separately in a room with temperature (22–24°C) and humidity (60-65%) on a 12h light/ dark cycle and fed with sufficient food and water. The experimental protocol was according to the National Institutes of Health Guide for the Care and Use of Laboratory Animals (National Institutes of Health Publication No. 85-23, revised 1996) and approved by the Committee of Experimental Animals of the First Affiliated Hospital of Shihezi University.

### 2.2 Animal model

C57BL/6 mice were injected intraperitoneally with LPS at a dose of 10 mg/kg (Sigma, USA) to induce sepsis (24). In order to inhibit miR-181a-5p or SIRT1, antagomirmiR-181a-5p (miR-181a-5p inhibitor), inhibitor control (GenePharma, Shanghai, China), control-siRNA (cat no. sc-36869; Santa Cruz Biotechnology, Inc., USA) or SIRT1-siRNA (cat no. sc-40986; Santa Cruz Biotechnology, Inc.) were injected by caudal vein, followed by LPS injection after 24 h of the last injection. Mice were only injected with LPS with a dose of 10 mg/kg in the LPS group. In the LPS plus inhibitor control group, mice were injected with an inhibitor control (2 mg/kg; 5′-UCACAACCUCCUA-GAAAGAGUAGA-3′) by tail intravenous injection 24 h prior to LPS treatment. In the LPS plus miR-181a-5p inhibitor group, mice were injected with the miR-181a-5p inhibitor (2 mg/kg; 5′-ACUCACCGACAGCGUUGAAUGUU-3′) by tail intravenous injection 24 h prior to LPS treatment. In the LPS plus control-siRNA group, mice were injected with control-siRNA (2 mg/kg) by tail intravenous injection 24 h prior to LPS treatment. In the LPS plus SIRT1-siRNA group, mice were injected with SIRT1-siRNA (2 mg/kg) by tail intravenous injection 24 h prior to LPS treatment. In the LPS plus miR-181a-5p inhibitor plus SIRT1-siRNA, mice were given the miR-181a-5p inhibitor (2 mg/kg) and SIRT1-siRNA (2 mg/kg) before LPS treatment. In the LPS plus inhibitor control group, mice were given an inhibitor control (2 mg/kg) before LPS treatment. 72 h later, the mice were killed through cervical dislocation (mice without a heartbeat and breathing were confirmed as dead) after anesthesia with pentobarbital (30 mg/kg, intraperitoneal injection). After sacrificing, the serum was detected and liver and kidney tissues were collected for subsequent analysis. The health and behavior of the mice were monitored every two days, and no mice died during our experiment. Throughout the whole experiment, we tried our best to alleviate the pain of the mice, and experiments were ended when the mice lost more than 15% of their body weight. Our experiment lasted for 4 days in total.

### 2.3 ELISA and biochemical marker detection

72 h after LPS treatment, the blood was collected from each sample, centrifuged at 3000 rpm for 15 minutes and then we collected the serum for biochemical analysis. The levels of pro-inflammatory cytokines (IL-1β, TNF-α and IL-6) were detected by using ELISA kits (Nanjing Jiancheng, China) according to the manufacturer’s instructions of each kit. In order to evaluate organ function, ALT, AST, Cr and BUN (Nanjing Jiancheng, China) levels in the serum of mice were measured according to the manufacturer’s instructions.

### 2.4 Cell culture and LPS treatment

The macrophage cell line RAW 264.7 was maintained in DMEM medium (Gibco, USA) supplemented with 10% fetal bovine serum (FBS, Gibco, USA), 10 U/ml penicillin-G, and 10 mg/ml streptomycin (Beyotime, Shanghai, China), and the cells were incubated at 37°C in a humidified atmosphere with 5% CO_2_. In the present research, RAW 264.7 macrophages were stimulated by 1 μg/ml LPS for 4 h (24), then the cells were harvested for subsequent experiments.

### 2.5 Cell transfection and reagents

miR-181a-5p inhibitor (5′-ACUCACCGACAGCGUUGAAUGUU-3′) and inhibitor control (5′-UCACAACCUCCUA-GAAAGAGUAGA-3′) were purchased from GenePharma (Shanghai, China). Before transfection, the cells were substituted in an antibiotic-free medium for 24 h, then inhibitor control, miR-181a-5p inhibitor, control-siRNA (cat no. sc-36869; Santa Cruz Biotechnology, Inc., USA), SIRT1-siRNA (cat no. sc-40986; Santa Cruz Biotechnology, Inc.) or miR-181a-5p inhibitor+SIRT1-siRNA were transfected into RAW 264.7 macrophages using Lipofectamin™ 2000 reagent (Invitrogen, USA) following the manufacturer’s instructions. qRT-PCR and western blot analysis were used to measure the efficiency of cell transfection.

### 2.6 Dual-luciferase reporter assay

We used target prediction databases (TargetScan) to predict the relationship between miR-181a-5p and SIRT1. Our results suggested that SIRT1 contains the target site of miR-181a-5p. Then, the SIRT1 3’-UTR, containing wild-type (WT) or mutant (MUT) target sites for miR-181a-5p were amplified by PCR and inserted into a pGL3-Control Vector (Promega, USA) to form the reporter vector SIRT1-wild-type (SIRT1-WT) or SIRT1-mutated-type (SIRT1-MUT) respectively. For luciferase reporter analysis, RAW 264.7 cells were co-transfected with luciferase reporter vectors, mimic control (5′-UCACAACCUCCUAGAAAGAGUAGA-3′) or miR-181a-5p mimic (5′-AACAUUCAACGCUGUCGGU-GAGU-3′) (GenePharma, Shanghai, China) by using lipofectamine 2000 (Invitrogen) for 48 h. Luciferase activity was detected by a Dual-Luciferase Reporter Assay System (Promega) according to the manufacturer’s protocol. Each experiment was repeated three times.

### 2.7 Quantitative real-time PCR (qRT-PCR) analysis

Total RNA from cells or tissues was extracted using Trizol reagent (Invitrogen, Carlsbad, CA, USA) according to the manufacturer’s instructions. In order to detect the mRNA level, PrimeScript™ RT reagent Kit (Takara, Japan) was used to transcribe 200 ng RNA to cDNA synthesis. Prism 7000 Real-Time PCR system (Applied Biosystems, USA) with SYBR Premix Ex Taq™ (TaKaRa, Shiga, Japan) were performed to measure the expressions of miR-181a-5p and SIRT1. GAPDH and U6 were performed as the internal controls. The cycle amplification conditions were as follows: 35 cycles of denaturing at 95˚C for 15sec, annealing at 60˚C for 60sec, chain extension step at 72˚C for 60sec and the final extension at 72˚C for 10 min. Primers were purchased from Sangon Biotech (Shanghai, China) and listed as following:

U6-forward, 5’GCTTCGGCAGCACATATACTAAAAT3’

reverse, 5’CGCTTCACGAATTTGCGTGTCAT3’;

GAPDH-forward, 5’CTTTGGTATCGTGGAAGGACTC3’;

reverse, 5’GTAGAGGCAGGGATGATGTTCT3’;

SIRT1-forward, 5’AATCCAGTCATTAAAGGTCTACAA3’;

reverse, 5’TAGGACCATTACTGCCAGAGG3’;

miR-181a-5p-forward, 5’CCGCGAACATTCAACGCTGTCG3’;

reverse, 5’ATCCAGTGCAGGGTCCGAGG-3’. The relative expression of target gene were calculated by relative quantification (2^−ΔΔCt^) method (25).

### 2.8 Western blot assay

The total proteins were extracted from cells using RIPA buffer (Auragene, Changsha, China). BCA Protein Assay Kit (Dingguo, Beijing, China) was performed to detect the protein concentration according to the manufacturer’s instructions. Then, the samples were mixed with 5× loading buffer, boiled at 100 °C for 5 min and separated by 10 % SDS-PAGE (Bio-Rad, Hercules, CA) and transferred onto PVDF membranes (Millipore, USA). After being blocked with 5% non-fat in 0.1% tris-buffered saline with Tween-20 for 1 h at room temperature, the membranes were incubated with antibodies at 4 °C overnight respectively with the following primary antibodies: p-p65 (dilution ratio: 1:1000; Cat no. ab86299; Abcam, MA, USA), p65 (dilution ratio: 1:1000; Cat no. ab16502; Abcam, MA, USA), SIRT1 (dilution ratio: 1:1000; Cat no. ab110304; Abcam, MA, USA) and β-actin (dilution ratio: 1:2000; Cat no. Ab8227; Abcam, MA, USA). After that, the membranes were washed with PBST four times and then incubated with a secondary antibody (Cat no. 7074; dilution ratio: 1: 2000; Cell Signaling Technology Inc., Danvers, MA, USA) for 1 h at room temperature. Finally, the protein bands were visualized by using an ECL detection system (Beyotime Institute of Biotechnology, China) according to the manufacturer’s instructions. Finally, protein bands were quantified by densitometry (QuantityOne 4.5.0 software; Bio-Rad Inc., USA).

### 2.9 Statistical analysis

All results were obtained from three independent experiments and were presented as mean±standard deviation (SD) Statistical analyses were calculated by using SPSS 16.0 software (IBM Corp., Armonk, NY). All experiments were performed in triplicate. Statistical significance was evaluated by a Student’s t-test, one-way or two-way analysis of variance (ANOVA) followed by Tukey’s post hoc test. The statistically significant level was set at p<0.05.

## 3 Results

### 3.1 MIR-181a-5p and inflammatory factors levels were significantly up-regulated in RAW 264.7 macrophages after LPS stimulation

**Figure 1:**
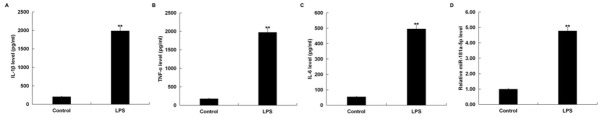
The levels of inflammatory factors and miR-181a-5p were increased in LPS induced RAW 264.7 macrophages. ELISA were used to detect the levels of IL-1β (A), TNF-α (B), and IL-6 (C) in RAW 264.7 macrophages with (LPS) or without (Control) LPS treatment. (D) miR-181a-5p expression in RAW 264.7 macrophages with (LPS) or without (Control) LPS treatment. The results were presented as the mean ± SD; **p<0.01 *vs*. Control.

1 μg/ml LPS was used to stimulate RAW 264.7 macrophages for 4 h. Then qRT-PCR was used to detect the level of miR-181a-5p, and ELISA was adopted to evaluate the expression level of IL-1β, TNF-α and IL-6. Our results indicated that the serum IL-1β, TNF-α and IL-6 levels significantly up-regulated in RAW 264.7 macrophages after LPS treatment compared with the control group (Figure 1A-1C). Moreover, the results from qRT-PCR assay showed that miR-181a-5p expression was significantly enhanced after LPS stimulation (Figure 1D).

### 3.2 SIRT1 was a direct target of miR-181a-5p and it was down-regulated in LPS induced RAW 264.7 cells

**Figure 2:**
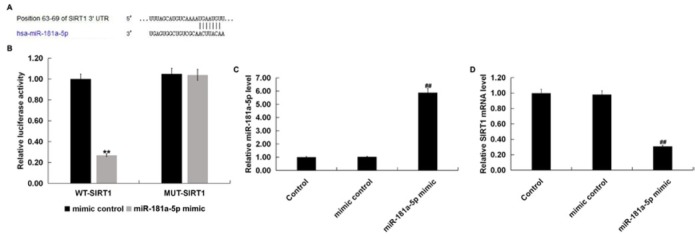
SIRT1 was a direct target of miR-181a-5p. The binding sites between miR-181a-5p and the 3′-UTR of SIRT1. (B) A dual-luciferase reporter assay was performed to measure the luciferase activities (**p<0.01 vs. mimic control). (C) the level of miR-181a-5p in RAW 264.7 macrophages transfected with mimic control or miR-181a-5p mimic was detected using qRT-PCR. (D) the mRNA level of SIRT1 in RAW 264.7 macrophages transfected with mimic control or miR-181a-5p mimic were detected using qRT-PCR. ##p<0.01 vs.Control

**Figure 3:**
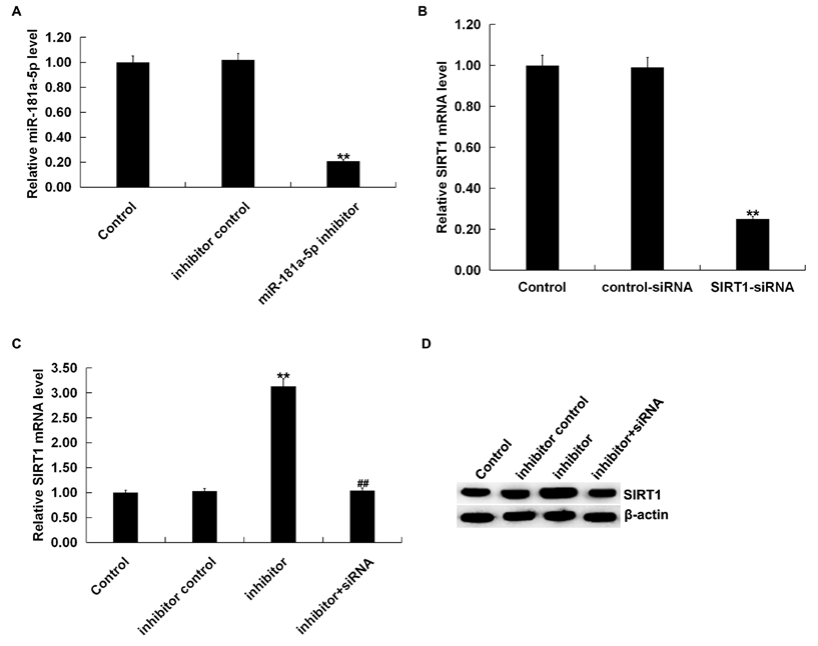
miR-181a-5p negatively regulated SIRT1 expression. (A) inhibitor control or miR-181a-5p inhibitor was transfected into RAW 264.7 macrophages for 48 h and qRT-PCR was carried out to detect the levels of miR-181a-5p. (B) control-siRNA or SIRT1-siRNA was transfected into RAW 264.7 macrophages for 48 h, then qRT-PCR was used to the detect the SIRT1 mRNA level. (C and D) 48 h after transfection with miR-181a-5p inhibitor, inhibitor control, or miR-181a-5p inhibitor+SIRT1-siRNA, the expression level of SIRT1 in RAW 264.7 macrophages was measured by qRT-PCR (C) and western blot (D) assay. Three independent assays were performed, and data were presented as mean±SD; **p<0.01 vs. Control; ##p<0.01 vs. inhibitor.

In order to better understand the molecular mechanisms of miR-181a-5p functions, a bioinformatics prediction tool (TargetScan) was used to identify the target sites between miR-181a-5p and SIRT1, and the results were presented in Figure 2A. A dual-luciferase reporter assay was constructed to further explore the specific relationship between miR-181a-5p and SIRT1. We found that up-regulated miR-181a-5p markedly decreased the luciferase activity of wild type SIRT1 3’UTR segment compared with the mimic control group, while the luciferase activity of SIRT1-MUT had no obvious decrease (Figure 2B). We also confirmed that miR-181a-5p mimic significantly enhanced miR-181a-5p level in RAW 264.7 macrophages (Figure 2C), and it significantly reduced SIRT1 mRNA expression (Figure 2D). Taken together, these results indicated that SIRT1 was a direct target of miR-181a-5p. Furthermore, a miR-181a-5p inhibitor, SIRT1-siRNA, and an inhibitor control or control-siRNA were transfected into RAW 264.7 macrophages for 48 h. As presented in Figure 3A, we found that the expression levels of miR-181a-5p were significantly decreased in cells transfected with miR-181a-5p inhibitor. Meanwhile, the relatively lower mRNA level of SIRT1 was observed in RAW 264.7 macrophages transfected with SIRT1-siRNA (Figure 3B). Moreover, the mRNA and protein levels of SIRT1 were remarkably advanced after miR-181a-5p inhibitor transfection, while the effects were reversed by SIRT1-siRNA transfection (Figure 3C and D). All these results indicated that miR-181a-5p negatively regulated the expression of SIRT1, which might affect the progression of sepsis.

### 3.3 The expression of SIRT1 was suppressed in LPS-activated RAW 264.7 macrophages

**Figure 4:**
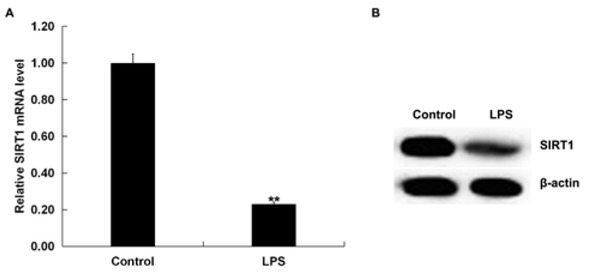
The level of SIRT1 was depressed in LPS-induced RAW 264.7 macrophages. qRT-PCR assay was carried out to elevate SIRT1 mRNA level in LPS-induced RAW 264.7 macrophages. (B) Western blot analysis was used to detect the protein level of SIRT1 in RAW 264.7 macrophages after LPS treatment. Data were expressed as mean±SD; **p<0.01 vs. Control.

RAW 264.7 macrophages were treated with LPS for 4 h. qRT-PCR and western blotting assays were adopted to detect the SIRT1 expression in LPS-activated macrophages and untreated macrophages. Our results showed that SIRT1 mRNA was significantly suppressed in the LPS group compared to the control group (Figure 4A). Consistently, SIRT1 protein expression was decreased in RAW 264.7 macrophages upon LPS treatment (Figure 4B).

### 3.4 MiR-181a-5p inhibitor decreased LPS-induced inflammatory factors expression in RAW264.7 macrophages by regulating SIRT1

**Figure 5:**
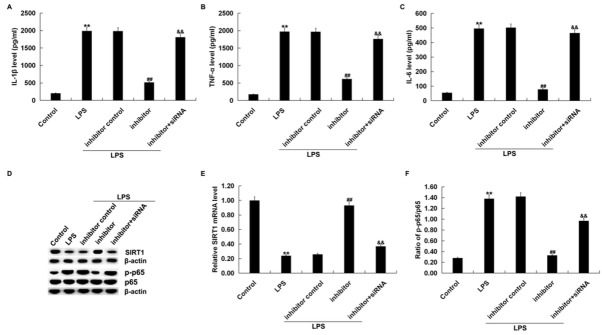
MiR-181a-5p inhibitor suppressed the LPS-induced inflammatory factors secretion and inhibited NF-κB activation. RAW 264.7 macrophages were transfected with inhibitor control, miR-181a-5p inhibitor or the miR-181a-5p inhibitor+SIRT1-siRNA for 48 h and then treated with 1 μg/ml LPS for 4 h. (A-C) The expression of inflammatory factors, including IL-1β, TNF-α and IL-6 were evaluated by ELISA. (D) The protein expression of SIRT1, p65 and p-p65 was measured by western blot assay. (E) The mRNA expression of SIRT1 was measured using qRT-PCR. (F) Ratio of p-p65/p65 was calculated and presented. Results were presented as mean± SD ; **p<0.01 vs. Control; ##p<0.01 vs. LPS; &&p<0.01 vs. inhibitor.

Previous studies have demonstrated that sepsis was associated with the inflammatory response. Therefore, RAW 264.7 macrophages were transfected with the inhibitor control, miR-181a-5p inhibitor or miR-181a-5p inhibitor+-SIRT1-siRNA for 48 h and then treated with 1 μg/ml LPS for 4 h. Then, the levels of IL-1β, TNF-α and IL-6 were measured by ELISA assay. We found that the secretion of IL-1β, TNF-α and IL-6 in RAW 264.7 macrophages were enhanced after treatment with LPS compared to the control group (Figure 5A-C). Meanwhile, compared to the LPS group, the miR-181a-5p inhibitor remarkably suppressed the secretion of TNFα, IL-1β and IL-6 that was stimulated by LPS treatment. In addition, compared with the miR-181a-5p inhibitor transfection group, the levels of IL-1β, TNF-α and IL-6 were significantly increased in SIRT1-siRNA co-transfection group (Figure 5A-C).

### 3.5 MiR-181a-5p inhibitor remarkably affected LPS-induced NF-κB activation by regulating SIRT1

We then further explored the underlying role of the miR-181a-5p inhibitor or SIRT1-siRNA on the signal pathway. RAW 264.7 macrophages were transfected with an inhibitor control, miR-181a-5p inhibitor or miR-181a-5p inhibitor+-

SIRT1-siRNA for 48 h, after that, we adopted 1 μg/ml LPS to stimulate the cells. Then, the protein level of p-p65 on the NF-kB pathway, SIRT1 mRNA and protein levels were detected after transfection. Results from western blotting assay and qRT-PCR analysis show that the reduced protein and mRNA levels of SIRT1 caused by LPS treatment were significantly enhanced by the miR-181a-5p inhibitor transfection, and this enhancement was reversed by SIRT1 silencing (Figure 5D and E). Besides this, the enhanced protein levels of p-p65 (Figure 5D) and increased p-p65/ p65 ratio (Figure 5F) caused by the LPS treatment were significantly decreased by the miR-181a-5p inhibitor, and this decrease was eliminated by SIRT1-siRNA. These data clearly demonstrated that miR-181a-5p was involved in the regulation of inflammatory response by targeting SIRT1 *in vitro*.

### 3.6 SIRT1-siRNA reversed the effects of miR-181a-5p inhibitor in LPS-induced septic mice

**Figure 6:**
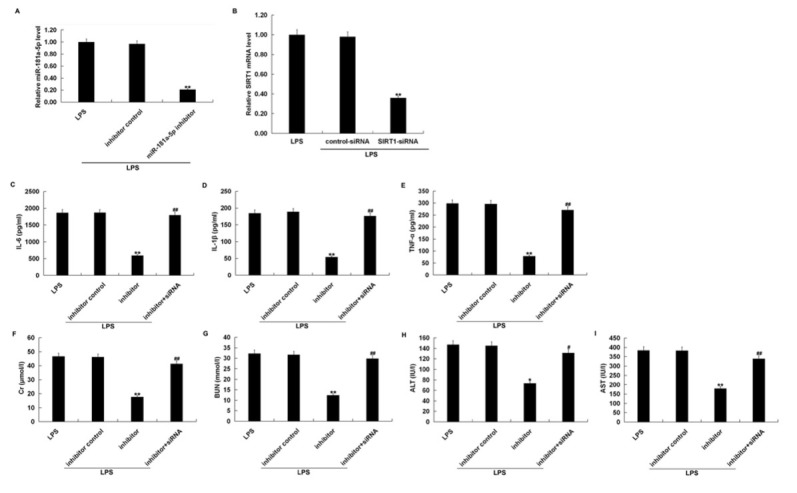
SIRT1-siRNA reversed the effects of miR-181a-5p inhibitor in LPS-induced septic mice. The level of miR-181a-5p was detected using qRT-PCR. (B) The mRNA level of SIRT1 was detected using qRT-PCR. (C-E) The IL-6, IL-1β, and TNF-α levels in the serum of mice in different groups were assessed by ELISA assay. (F-I) ELISA assay was carried out to evaluate the Cr, BUN, ALT and AST levels in serum of mice in different groups, respectively. Three independent experiments were performed. The results were expressed as mean±SD; *, **p<0.05, 0.01 vs. LPS; #, ##p<0.05, 0.01 vs. inhibitor.

To evaluate whether miR-181a-5p was related to an inflammatory response in septic mice, septic mice were injected with the inhibitor control, miR-181a-5p inhibitor or the miR-181a-5p inhibitor+SIRT1-siRNA. Our data show that compared with the LPS treatment alone group, the level of miR-181a-5p in mice treated with LPS plus miR-181a-5p inhibitor was significantly reduced (Figure 6A). The SIRT1-siRNA injection also significantly reduced the mRNA level of SIRT1 in mice treated with LPS (Figure 6B). Then, the levels of IL-6, IL-1β and TNF-α were measured by ELISA assay. As results showed in Figure 6C-E, the secretion of inflammatory factors, including IL-6, IL-1β and TNF-α, were significantly decreased in the LPS+miR-181a-5p inhibitor group compared with the LPS treatment group. However, in the LPS+miR-181a-5p inhibitor+SIRT1-siRNA group, the levels of IL-6, IL-1β and TNF-α were increased. Additionally, the infiltration of kidney or liver injury markers (Cr, BUN, ALT and AST) in the serum were notably alleviated in the miR-181a-5p inhibitor group, while these biological markers expression were remarkably increased in the LPS+miR-181a-5p inhibitor+SIRT1-siRNA group (Figure 6F-I). These data suggested that the miR-181a-5p inhibitor relieved the inflammatory response and kidney and liver damage through negatively regulating SIRT1 expression in septic mice.

## 4 Discussion

Previous studies have revealed that the occurrence and progression of sepsis is closely related to a dysregulated immune response [26]. The initial hyper-inflammatory state may develop into an immunosuppressive state [27]. Several studies have suggested that miRNAs play vital roles in the regulation of the immune response through influencing vital signaling elements. Recently, Zhu et al. found that oligochitosan up-regulated miR-146a, miR-181a, miR-181b, and miR-301a-3p expression, leading to reduced TNF-α expression in RAW 264.7 macrophages (28). In addition, Wang et al. have identified that up-regulation of miR-130b decreased serious lung inflammation in the sepsis mice model after LPS treatment [29]. miR-181a-5p belongs to the miR-181 family. Several reports have shown that miR-181a-5p was highlighted in various cells, such as HTR-8/SVneo cells, hepatocellular carcinoma and lung cancer cells [30, 31, 32]. Nevertheless, little is known about whether and how miR-181a-5p is involved in immune regulation in sepsis. Based on these researches, in this study, we elucidated the role of miR-181a-5p in the sepsis inflammatory response.

Firstly, RAW 264.7 macrophages were stimulated by 1 μg/ml LPS for 4 h. Then we adopted ELISA and qRT-PCR assays to evaluate the inflammatory factors (IL-1β, TNF-α and IL-6) level and miR-181a-5p level in the LPS treatment group and control group. Our results showed that IL-1β, TNF-α, IL-6 and miR-181a-5p levels were significantly up-regulated in LPS stimulated RAW 264.7 macrophages, which were in accordance with other relevant researches, suggesting that the inhibition of miR-181a-5p expression might block the development of inflammation and function as an anti-inflammatory role in diseases.

It has been reported that miR-181a-5p affects cell functions possibly by targeting SBP2, c-Met and IGF2BP2 [33, 34, 35]. Therefore, we used bioinformatic tools to reveal putative mRNA targets of miR-181a-5p. We confirmed that miR-181a-5p directly targeted SIRT1 and it negatively regulated the expression of SIRT1 in RAW 264.7 macrophages, thus revealing a possible mechanism of SIRT1 with inflammation response in sepsis. In order to verify the hypothesis, we investigated the roles of the miR-181a-5p inhibitor in LPS treated RAW 264.7 macrophages. Then, we used qRT-PCR and western blot analysis to measure the levels of SIRT1 in LPS-induced RAW 264.7 macrophages. Our present results indicate that SIRT1 was down-regulated in LPS stimulated RAW 264.7 macrophages.

SIRT1 was found to regulate the NF-κB signal pathway, and up-regulation of SIRT1 could inhibit NF-κB activation and affect cell growth [36]. Therefore, we further explored the roles of the miR-181a-5p inhibitor or SIRT1-siRNA on inflammatory factors including TNF-α, IL-1β, IL-6 and NF-κB signal relative genes (SIRT1 and p-p65). We observed that miR-181a-5p inhibitor alleviated the secretion of inflammatory factors in RAW 264.7 macrophages with LPS stimulation. Besides, compared to the control group, the expression of p-p65 was suppressed and SIRT1 level was increased in LPS-induced RAW 264.7 macrophages. These data suggested that miR-181a-5p was involved in the inflammatory progress by targeting SIRT1 to influence NF-κB signaling pathway in RAW 264.7 macrophages. However, in the present study, the nuclear translocation of p65, p-IkB, and p-IKK-beta protein expression was measured, and this may be an imitation of this study. In the future we will delve deeper into the effects of miR-181-5p on NF-κB signaling pathways in sepsis.

Previous researches focused on exploring the mechanism of miR-181a-5p *in vitro*. Then, we also determined the effects of miR-181a-5p inhibitor or SIRT1-siRNA in mice with sepsis induced by LPS administration. ELISA assay revealed that compared with the LPS treatment group, inflammatory factors (IL-1β, TNF-α, IL-6) levels and organ damage markers (ALT, AST, Cr and BUN) expressions were inhibited in the miR-181a-5p inhibitor+LPS group. Similar to the *in vitro* results, SIRT1-siRNA reversed these effects. In summary, the present study indicated the anti-inflammatory activity of the miR-181a-5p inhibitor both *in vitro* and *in vivo* inflammatory models. However, our study has a limitation, we did not set up the inhibitor+control-siRNA group.

In conclusion, our data demonstrated that miR-181a-5p was up-regulated in sepsis mice and LPS stimulated RAW 264.7 cells. miR-181a-5p down-regulation inhibited inflammatory response *in vitro* and *in vivo* through repressing NF-κB signaling pathway by targeting SIRT1, indicating the anti-inflammatory effect of miR-181a-5p inhibitor in sepsis. It was also the first time investigating the role of miR-181a-5p in sepsis. Currently, miRNAs have been shown to be bio-markers and therapeutic targets in a variety of diseases [37, 38, 39]. Some miRNAs have been shown to be abnormally expressed in sepsis, and changes in their expression may influence the pathology and progression of sepsis [14, 15, 16]. As, sepsis is a serious systemic inflammatory response syndrome [1, 2], our data indicated that miR-181a-5p is a potential therapeutic target for anti-inflammatory treatment [40, 41] of sepsis. However, this was just a preliminary study of miR-181a-5p in sepsis. To make the role of miR-181a-5p in sepsis more convincing, a lot of in-depth research is still needed. For instance, correlation analysis between miR-181a-5p and the SIRT1 gene and protein expression in macrophage cell line RAW264.7 should be made. The role of SIRT1 alone in sepsis *in vitro* and *in vivo* needs to be explored. Histological of macrophages in liver and in kidney of sepsis mice before and after miR-181a-5p inhibitor treatment should be investigated. The expression of miR-181a-5p/SIRT1 in patients with sepsis, and the relationship between the expression of miR-181a-5p/SIRT1 and the clinical features of patients with sepsis are necessary to be explored. We will study these issues in the future.
